# A systematic review and meta-analysis of gestational weight gain recommendations and related outcomes in Brazil

**DOI:** 10.6061/clinics/2015(11)08

**Published:** 2015-11

**Authors:** Ana Carolina Godoy, Simony Lira do Nascimento, Fernanda Garanhani Surita

**Affiliations:** I Universidade Estadual de Campinas (UNICAMP), Faculdade de Ciências Médicas, Departamento de Tocoginecologia, Campinas/SP, Brazil; II Universidade Federal do Ceará, Faculdade de Medicina, Departamento de Fisioterapia, Fortaleza/CE, Brazil

**Keywords:** Weight Gain, Body Mass Index, Pregnancy

## Abstract

Worldwide, different guidelines are used to assess the adequacy of gestational weight gain. This study identified the recommendations for gestational weight gain in Brazilian women. We also determined the proportion of women with adequate weight gain in accordance with these recommendations and the associated perinatal outcomes.

A systematic review was performed. A computerized search was conducted utilizing the following databases: PubMed, MEDLINE, Web of Science, Embase, SciELO and Google Scholar. Observational studies of healthy, Brazilian, pregnant women were included. Studies were excluded if they did not provide pregestational weight and gestational weight gain or if they studied women with comorbid conditions. A meta-analysis was performed to evaluate the odds ratio of inadequate (insufficient or excessive) gestational weight gain.

Seventeen studies were included in the systematic review and four studies were included in the meta-analysis. The most widely used recommendations were from the Institute of Medicine. Excessive gestational weight gain was associated with fetal macrosomia and high rates of cesarean delivery. Overweight women had a higher risk of excessive gestational weight gain than eutrophic women (OR=2.80, 95%CI=2.22-3.53).

There are no standardized recommendations concerning gestational weight gain based on Brazilian population-based data. Many Brazilian women are overweight or obese at the beginning of pregnancy. Overweight pregnant women have a higher risk of excessive gestational weight gain. Excessive gestational weight gain was associated with cesarean delivery and fetal macrosomia.

## INTRODUCTION

A high preconception body mass index (BMI) and excessive gestational weight gain (GWG) may be associated with adverse perinatal outcomes 1.

GWG results from diverse structural and functional modifications that occur in a woman's body to meet the nutritional demands of pregnancy. The increase in weight is due to diverse factors, such as the fetus, amniotic fluid, placenta, increased blood volume, increased adipose tissue and uterine and mammary growth 2. Using this knowledge, recommendations for GWG were created.

There are a variety of recommendations for different populations 4. One recommendation is Atalah's curve, a graph that allows monitoring of the progress of nutritional status during pregnancy based on pregestational BMI. This curve was created based on data from Chilean women 3. In the United States, the recommendations are issued by the Institute of Medicine (IOM) and are based on a woman's pregestational BMI; the IOM recommendations estimate a range of weight gain per pregnancy trimester 5-6.

Recommendations by the Brazilian Ministry of Health are based on an amalgamation of data from Atalah's curve and the IOM recommendations. There is a range of expected weight gain for each gestational week and this range is based on the pregestational BMI. A graph shows the following four ranges that classify nutritional status during pregnancy: underweight, adequate weight, overweight and obese.

It is estimated that 50% of reproductive-aged women are overweight or obese and 18% of these women are already overweight or obese when they become pregnant 7. According to the World Health Organization (WHO), the prevalence of obesity during pregnancy ranges from 1.8% to 25.3% and is also related to increased maternal-fetal risk 8. For these women, guidance about GWG is essential during prenatal care.

Despite recommendations, a large number of pregnant women have excessive weight gain 9-10. Brazilian women who start prenatal care in public health services are not eutrophic in early pregnancy 11.

Excessive GWG is associated with maternal and fetal complications, such as gestational diabetes, gestational hypertension, preeclampsia, labor induction, cesarean section, anesthetic complications, postpartum hemorrhage, stillbirth, macrosomia, NICU admission, prematurity, congenital abnormalities and childhood obesity with long-term problems 1,12.

Low maternal weight and insufficient GWG also need attention and may be associated with fetal growth restriction, low birth weight and prematurity 13.

We conducted a systematic review of the literature to determine GWG recommendations for Brazilian women. We also determined the proportion of women who had adequate weight gain according to these recommendations and their association with perinatal outcomes.

## MATERIALS AND METHODS

### Data sources and searches

A systematic search of the following databases was conducted to identify relevant studies: MEDLINE, PubMed, EMBASE, SciELO, Web of Science and Google Scholar. The MeSH search terms included: (“gestational weight gain”) AND (“gestation” OR “pregnancy”) AND (“Brazil”). The search strategy was designed for the PubMed database and altered as needed for use in other databases.

### Study selection and data extraction

The following inclusion criteria were considered: observational studies (cross-sectional and cohort); conducted in Brazil and published in English, Portuguese or Spanish; no date limit of publication; and studied healthy pregnant women in any BMI category. Studies with results showing the average GWG or the proportion of adequacy to recommended GWG were included. Studies that performed any intervention, included women with specific comorbid conditions, or specifically assessed adolescents were excluded.

### Data collection and analysis

The study search and screening were completed independently by two reviewers. All articles identified were screened by reading the respective titles and abstracts. Non-original articles, review articles and articles without data regarding pregestational and gestational weight or articles describing comorbid conditions were excluded. The remaining articles were fully assessed by two independent researchers for evaluation. After their assessments, these researchers compared their results. Discrepancies were resolved by consensus. When necessary, a third senior evaluator decided whether to include an article. Studies showing pregestational BMI ranges, GWG or average GWG were included. The reference lists of the remaining articles were manually checked to identify additional studies.

A meta-analysis was performed for studies with complete data on the adequacy of GWG according to the pregestational BMI categories (classified as insufficient, adequate or excessive). The meta-analysis determined the odds ratios for insufficient and excessive GWG according to the pregestational BMI categories. Three groups were established for comparison, using eutrophic pregnant women as a reference: 1) underweight *vs*. eutrophic; 2) overweight *vs*. eutrophic; and 3) obese *vs*. eutrophic. The outcomes evaluated the odds ratios for excessive and insufficient GWG in each comparison group. The number of events (excessive or insufficient GWG), the total number of pregnant women in each category (underweight, overweight or obese) *versus* the number of events (adequate GWG) and the total number of eutrophic pregnant women were extracted from each study. These data were then used to generate odds ratios and confidence intervals for each study. The combination of study results included in the meta-analysis generated a final odds ratio for each analysis. The Mantel-Haenszel test with a fixed-effects model was used to evaluate the significance of the results. A forest plot graph was generated for each analysis. All analyses were performed in Review Manager **(RevMan, version 5.3, The Cochrane Collaboration, Oxford, UK)** 14.

To evaluate the methodological quality of the observational studies included in this review, the Strengthening the Reporting of Observational Studies in Epidemiology (STROBE) Statement was used based on the STROBE 2007 Checklist 15. The checklist contains 22 items that allow readers to understand the methods, analysis and validity of the results shown by the studies. To conduct this review, the criteria of the “Meta-analysis of Observational Studies in Epidemiology (Moose) Statement” were used 16.

This study complied with all the recommended ethical principles and confidentiality of information guidelines. Formal approval from a Research Ethics Committee was not required because the study was an analysis of results that had already been published in other articles in the public domain. This project was recorded in a database of systematic reviews -PROSPERO (Prospective International Record of Systematic Review) - PROSPERO 2013:CRD42013004366.

## RESULTS

**Figure 1:**
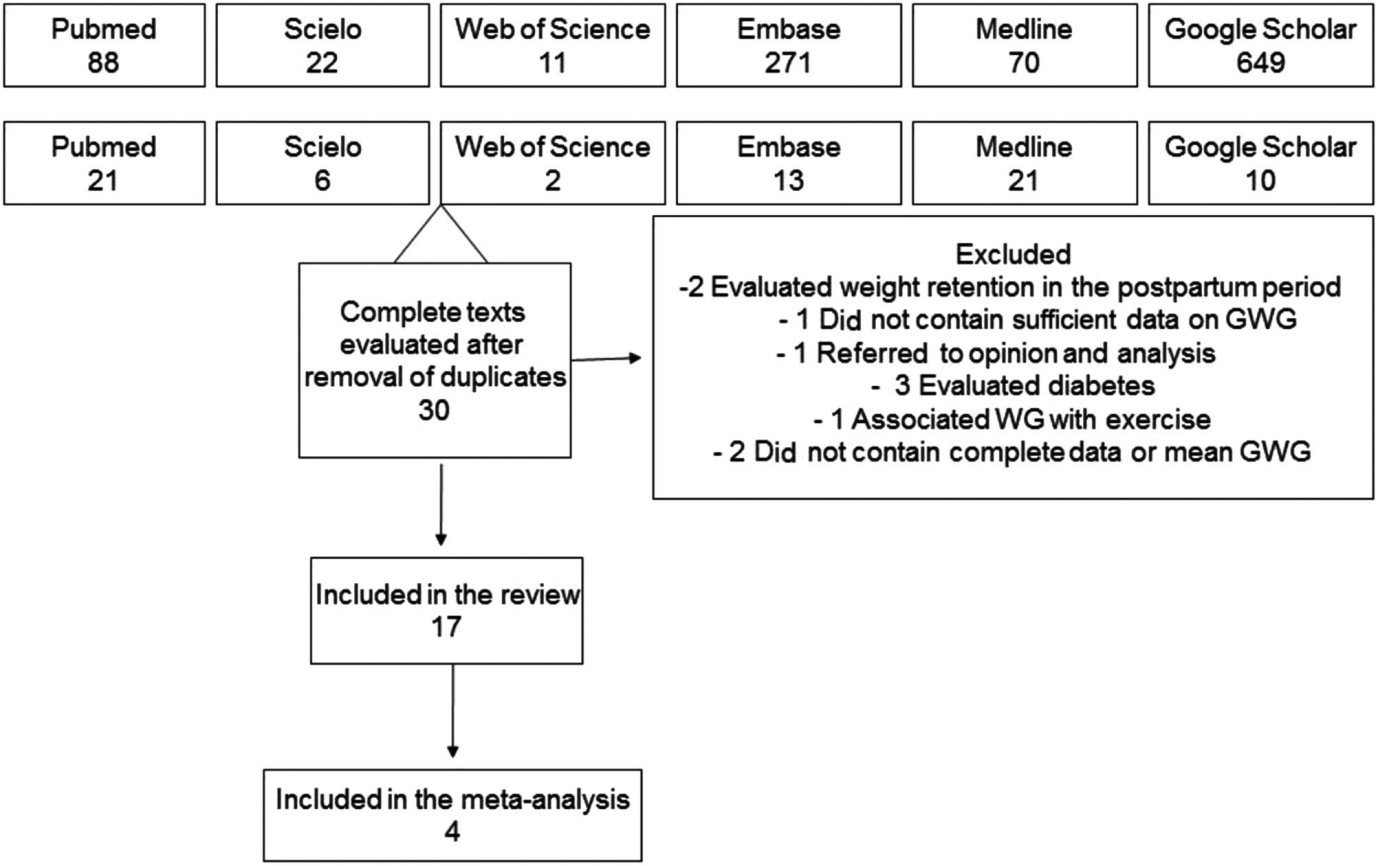
Flow chart.

A total of one thousand one hundred eleven (1,111) articles were identified by keywords in a computerized search. After reading the titles and abstracts, 73 articles remained. Of these remaining articles, duplicate articles were removed, resulting in 30 articles. In total, 17 articles were selected and included in the systematic review (Figure 1).

Table 1 describes the characteristics and main results of the 17 studies included in this review.

Regarding the Brazilian regions where the studies were conducted, the majority were conducted in the Southeast (17-20,22,23,26,28,30,32) and Northeast (24,29,31). The most commonly used recommendations were those of the IOM (13/17).

Four studies that had complete data for GWG and adequacy per BMI category used the IOM recommendations (17,20,22,26). In three of these studies, more than 50% of obese and overweight women had excessive GWG (17,20,22) (Table 2).

Concerning the influence of maternal weight on neonatal outcomes, two studies found an association between excessive GWG and low neonatal birth weight 20,. One study found an association between overweight/obesity and fetal macrosomia as well as between overweight/obesity and a higher risk of premature delivery 17. Three studies identified an association between higher rates of cesarean section and excessive GWG (17,20,23).

**Figure 2:**
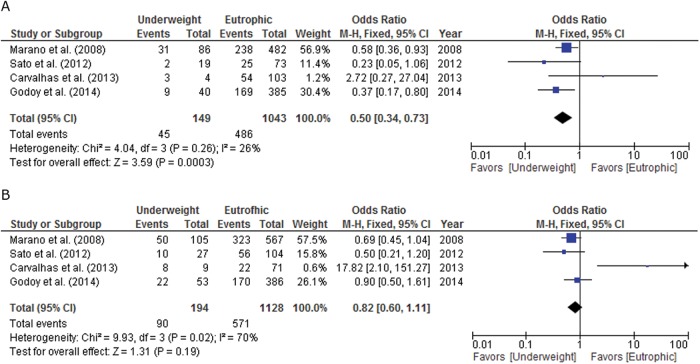
Forest plot showing the odds of gaining excessive (A) and inadequate (B) weight among underweight pregnant women compared to eutrophic pregnant women.

**Figure 3:**
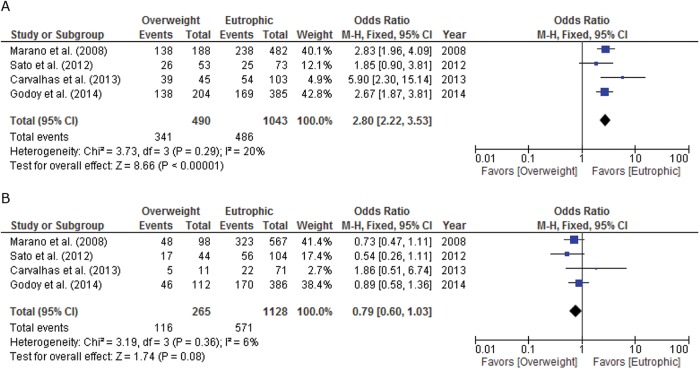
Forest plot showing the odds of having excessive (A) and insufficient (B) weight among overweight pregnant women compared to eutrophic pregnant women.

**Figure 4:**
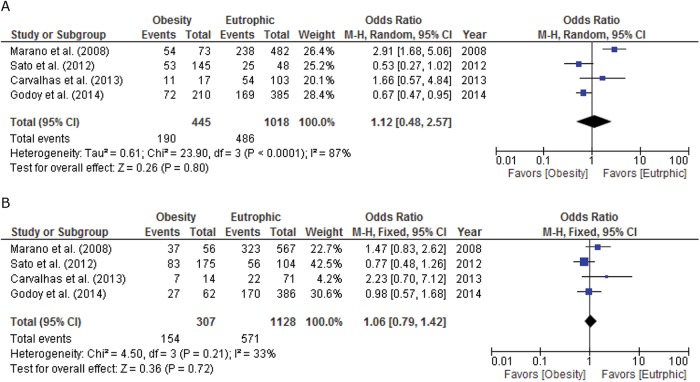
Forest plot showing the odds of gaining excessive (A) and insufficient (B) weight among obese pregnant women compared to eutrophic pregnant women.

When comparing underweight, overweight and obese with eutrophic pregnant women, the meta-analysis showed that underweight women had a lower chance of excessive GWG (Figure 2). Overweight women had a higher chance of excessive GWG (Figure 3). Pregnant women with pregestational obesity showed no difference regarding the odds of having excessive or insufficient GWG (Figure 4).

All 17 studies included in the review were submitted for evaluation of methodological quality through the STROBE 2007 Checklist 15 and none fulfilled all of the criteria. These recommendations must be followed to facilitate a comparison between studies and improve the methodological quality of observational studies. The least followed criteria were as follows: the study design did not appear in the title 18,, the sample size calculation was not specified (19,21,22,25,24,28,30-32) and there was no mention of financial support 17,18,22,24,25,26,29,31,32.

## DISCUSSION

The recommendations for GWG that are most commonly used in Brazil are based on the IOM. There are two versions of these recommendations: 1990 and 2009. The most recent version added a range of GWG for obese women 6.

The IOM recommendations are based on North American population data, which limits its use in populations with diverse ethnic characteristics and nutritional habits. However, the IOM recommendations are widely used in many countries, including Brazil, mainly for research purposes because it provides a lower and upper threshold of GWG for each pregestational BMI category.

Difficulties in standardizing the recommendations for GWG have also been reported in other studies. A review of the literature on nutritional status in Brazilian women (until the year 2007) found that inadequate anthropometric methods were used for gestational evaluations. A specific curve for GWG with national data should be generated to help standardize recommendations 35.

A descriptive study with 240 pregnant Brazilian women described difficulty in choosing the best method to assess nutritional status during pregnancy. The optimal method is currently a topic of great discussion among the literature and among entities responsible for nutritional monitoring in Brazilian health care services 36.

The recommendations used for weight gain adjustment in pregnant women in the United States, which are clearly based on the IOM, provide guidelines for GWG based on the optimization of short-term and long-term maternal and infant health outcomes 36. Australia also uses the IOM recommendations. However, in a study performed with 1059 women, when asked at 16 weeks of gestation, 47% of the women were skeptical of the GWG recommendations that they received. Furthermore, 62% of these women reported having never or rarely received instructions for GWG from health care professionals during their prenatal care. In conclusion, the majority of these women did not know or receive any GWG recommendations 37.

### Adequacy of GWG in Brazilian pregnant women

The main outcome of this review shows a higher risk of excessive GWG for pregestational overweight women (OR= 2.80 [2.22, 3.53]). This review also found that the overweight and obese indexes are elevated in women of reproductive age who become pregnant in Brazil.

A study investigating 204 pregnant women in the Northeast region of Brazil showed that 34.6% of women began pregnancy with a high BMI (overweight and obese) and 45.5% experienced excessive GWG 24. In another study in the Southern region, the prevalence of excessive GWG was 46.5% and 45.9% among overweight and obese women, respectively 21.

Obese women have become the focus of interventions and concern by health care professionals. However, overweight women have the highest risk and the highest rates of excessive GWG. Therefore, these women need more attention and specific interventions by health professionals because this group appears to be unnoticed. Thus, this group is at risk of becoming obese after pregnancy 17.

A study conducted in the Brazilian Southeast showed that the initial nutritional status in the majority (59.0%) of pregnant women was eutrophic and 29.7% had adequate GWG 17. Another study observed 1051 pregnant women and found that 38.9% of eutrophic women had adequate GWG and showed that a normal BMI at the beginning of pregnancy was often associated with an adequate GWG 17. Regarding insufficient GWG, the same study showed that 26.6% had insufficient GWG, which is alarming and may be a specific problem in some populations 17.

### Perinatal results associated with GWG

An association between macrosomia and pregestational overweight/obese women was observed in studies from the Northeast and Southeast of Brazil 17,29,32. In one of these studies, women with excessive GWG had a greater chance of having a macrosomic newborn 17. In another study, the incidence of macrosomia was 4.8% and 10.4% for overweight and obese women, respectively 32.

Not only do excessive weight and obesity influence perinatal results but insufficient GWG may also lead to complications, such as low birth weight and fetal growth restriction. Women with low pregestational body weight had a higher chance of having infants with low birth weights 28.

The mode of delivery may also be influenced by maternal weight and there are some concerning data. In a study performed with 204 pregnant women in the Northeast, high pregestational BMI ≥25 kg/m^2^ was an independent predictor of cesarean delivery 24.

Another prenatal outcome that is influenced by GWG is the occurrence of premature delivery. In a study performed with 212 women, 5.7% of the infants were premature, although there was no association between excessive GWG and preterm delivery 20. A study with 1052 women observed that the prematurity rate was higher among obese pregnant women than underweight pregnant women 17. In contrast, in a study performed in the South, the risk of premature delivery was higher among women whose weight gain was ≤8 kg, regardless of pregestational BMI. The prematurity rate observed among women with excessive GWG deserves special attention because it could be related to therapeutic premature delivery due to the increased morbidity associated with obesity and excessive GWG 38.

### GWG and weight retention during the postpartum period

For reproductive-aged women, excessive GWG with weight retention during the postpartum period increases the risk of obesity. Furthermore, excessive weight increases the risk of developing preeclampsia, gestational hypertension, cesarean section, premature delivery and fetal macrosomia in future pregnancies. A study performed with 715 pregnant women evaluated the risk of excessive weight during the postpartum period found that the prevalence of excessive weight 12 months after delivery was higher than during the gestational period and 30.7% of the women retained more than 10 kg. Furthermore, 12 months after delivery, weight retention was greater in women who were overweight during the pregestational period compared with eutrophic women 21.

Brazil is a large developing country, and most women are of reproductive age. Despite a drop in the fertility rate, the absolute number of births is still high in Brazil. Therefore, specific recommendations are needed for adequate GWG due to the impact on neonatal outcomes and the woman's future health.

There are no standardized recommendations concerning GWG based on Brazilian population-based data. The most commonly used recommendations are the IOM recommendations.

A large proportion of Brazilian women are overweight or obese at the beginning of pregnancy. Overweight pregnant women have a higher risk of excessive GWG.

Excessive GWG was associated with cesarean delivery and fetal macrosomia.

Guidance about adequate GWG and strategies for stimulating physical activity and nutritional guidance during pregnancy are fundamental tools that can decrease the risk of weight retention during the postpartum period and future obesity.

## Figures and Tables

**Table 1:** Characteristics and main results of the 17 studies included in this review.

Author/Year	Study	Number of women/City/Region	Adopted recommendation	Results
Godoy, 2014^(17)^	Cross-sectional	1052 – Campinas – Southeast	IOM, 2009	Mean WG: 13.08 kg. Obese (13.6%) and overweight (24.6%) women were 55.9% and 53.7% of the EWG, respectively.
Fraga, 2014^(18)^	Cross-sectional	1079 – Rio de Janeiro- Southeast	IOM, 1990	Mean WG: 12.3 kg. 30% of pregnant women had appropriate weight gain during pregnancy. 50% had EWG.
Fonseca, 2013^(19)^	Cross-sectional	712- Jundiaí – Southeast	MH, 2004	Mean WG: 13.20 kg. 34% of pregnant women were obese or overweight according to their BMIs in early pregnancy.
Carvalhaes, 2013^(20)^	Cross-sectional	212 - Botucatu – Southeast	IOM, 2009	Pre-pregnancy BMI: 59% adequate, 23.6% overweight and 11.8% obese. EWG were 50.5%, 29.7% AWG, and 19.8% of the IWG. Among overweight women, 78% showed EWG.
Nast, 2013^(21)^	Longitudinal	715- Porto Alegre- South	Atalah, 1997	Mean WG: 11.6 kg. 46.5% among those with OW, 45.9% among those with OB and 17.6% were between eutrophic and EWG.
Marano, 2012^(22)^	Descriptive	1287- Rio de Janeiro- Southeast	IOM, 2009	Pre-pregnancy weight, 26.6% overweight or obese and 11% underweight. 35.6% had EWG and 35.8% IWG. A low pre- pregnancy weight was protective against EWG.
Fernandes, 2012^(23)^	Cross-sectional	592-Rio de Janeiro- Southeast	IOM, 2009	Pre-pregnancy weight: Adequate-64.9%, 22.3%, Overweight, obesity, 12.8%. 39.5% had EWG.
Santos, 2012^(24)^	Descriptive	204 – Salvador – Northeast	IOM, 1990	34.6% had higher pre-pregnancy BMIs. 45.5% had excessive EWG.
Gonçalves, 2012^(25)^	Cross-sectional	1235- Rio Grande - South	IOM, 2009	Mean pre-pregnancy weight: 63.6 kg. Mean weight in late pregnancy: 73 kg. Mean weight gain during gestation: 9.4 kg.
Sato, 2012^(26)^	Retrospective	228 - São Paulo – Southeast	MH, 2004	30% initial BMI obese and overweight. 37.1% of obese and overweight had EWG.
Drehmer, 2010^(27)^	Cross-sectional	667 – Porto Alegre – South	IOM, 2009	Insufficient WG: 25.8%. 44.8%: excessive. For women with less than 6 prenatal visits, 52% had a higher risk of insufficient WG.
Padilha, 2009^(28)^	Cross-sectional	433 – Rio de Janeiro – Southeast	IOM, 1990	64.8% normal pre-pregnancy weight. Total mean WG: 12.99 kg.
Amorin, 2009^(29)^	Cross-sectional	551 – Campina Grande – Northeast	IOM, 1990	Mean WG: 11.4 kg. EWG in 21.3%, 35.4% AWG in women.
Rodrigues, 2008^(30)^	Cohort	225 – Rio de Janeiro- Southeast	IOM, 1990	Mean pre-pregnancy weight: 61,2 kg. Mean Total WG: 11.7 kg.
Andreto, 2006^(31)^	Descriptive	240–Recife- Northeast	Atalah, 1997	48.3% entering pregnancy had a normal weight, and 26.3% were overweight or obese. Excessive weight gain in the 2nd quarter was higher among overweight and obese women (6.3%).
Kac, 2005^(32)^	Cohort	230 – Rio de Janeiro - Southeast	IOM, 1990	Excessive WG 29.1%, 34.4% AEG and 36.5% IWG
Nucci, 2001^(33)^	Cohort	3082 - 6 cities (Southeast, South, Northeast)	IOM, 1990	38% had IWG, and 29% had EWG.

a) WG: weight gain; b) EWG: excessive weight gain; c) kg: kilograms; d) AWG: adequate weight gain; e) IWG: insufficient weight gain; f) OW: overweight; g) OB: obese; BMI: body mass index; MH: ministry of health.

**Table 2:** Comparison of gestational weight gain based on pregestational Body Mass Index from four studies included in the meta-analysis (all studies used the Institute of Medicine recommendations).

Author/Year/Number of women included	Insufficient weight gain n (%)	Adequate weight gain n (%)	Excessive weight gain n (%)
**Godoy, 2014^(17)^** **n=1052**	UW 22 (35.5)	UW 31 (50.0)	UW 9 (14.5)
	EU170 (30.6)	EU 216 (38.9)	EU 169 (30.5)
	OW 46 (18.6)	OW 66 (25.5)	OW 138 (55.9)
	OB 27 (20.1)	OB 35 (26.1)	OB 72 (53.7)
**Carvalhaes, 2013^(20)^** **n=212**	UW 8 (66.7)	UW 1 (8.3)	UW 3 (25)
	EU 22 (17.6)	EU 49 (39.2)	EU 54 (43.2)
	OW 5 (10.0)	OW 6 (12.0)	OW 39 (78.0)
	OB 7 (28.0)	OB 7 (28.0)	OB 11 (44.0)
**Sato, 2012^(26)^** **n=228**	UW 10 (34.5)	UW 17 (58.6)	UW 2 (6.9)
	EU 56 (43.4)	EU 48 (37.2)	EU 25 (19.4)
	OW 17 (24.3)	OW 27 (38.6)	OW 26 (37.1)
	OB 83 (36.4)	OB 92 (40.3)	OB 53 (23.2)
**Marano, 2012^(22)^** **n=1287**	UW 50 (37.0)	UW 55 (40.0)	UW 31 (23.0)
	EU 323 (40.0)	EU 244 (30.0)	EU 238 (30,0)
	OW 48 (20.0)	OW 50 (21,0)	OW 138 (59.0)
	OB 37 (34.0)	OB 19 (17.0)	OB 54 (49.0)

a) IOM: Institute of Medicine; b) BMI: Body Mass Index; c) UW: Underweight; d) EU: Eutrophic; e) OW: Overweight; f) OB: Obese.
